# A Stiff, Tough, and Thermally Insulating Air- and Ice-Templated Plant-Based Foam

**DOI:** 10.1021/acs.biomac.2c00313

**Published:** 2022-05-27

**Authors:** Tamara L. Church, Konstantin Kriechbaum, Carina Schiele, Varvara Apostolopoulou-Kalkavoura, Seyed Ehsan Hadi, Lennart Bergström

**Affiliations:** † Department of Materials and Environmental Chemistry, Stockholm University, Stockholm 10691, Sweden; ‡ Wallenberg Wood Science Center, Department of Materials and Environmental Chemistry, Stockholm University, Stockholm 10691, Sweden

## Abstract

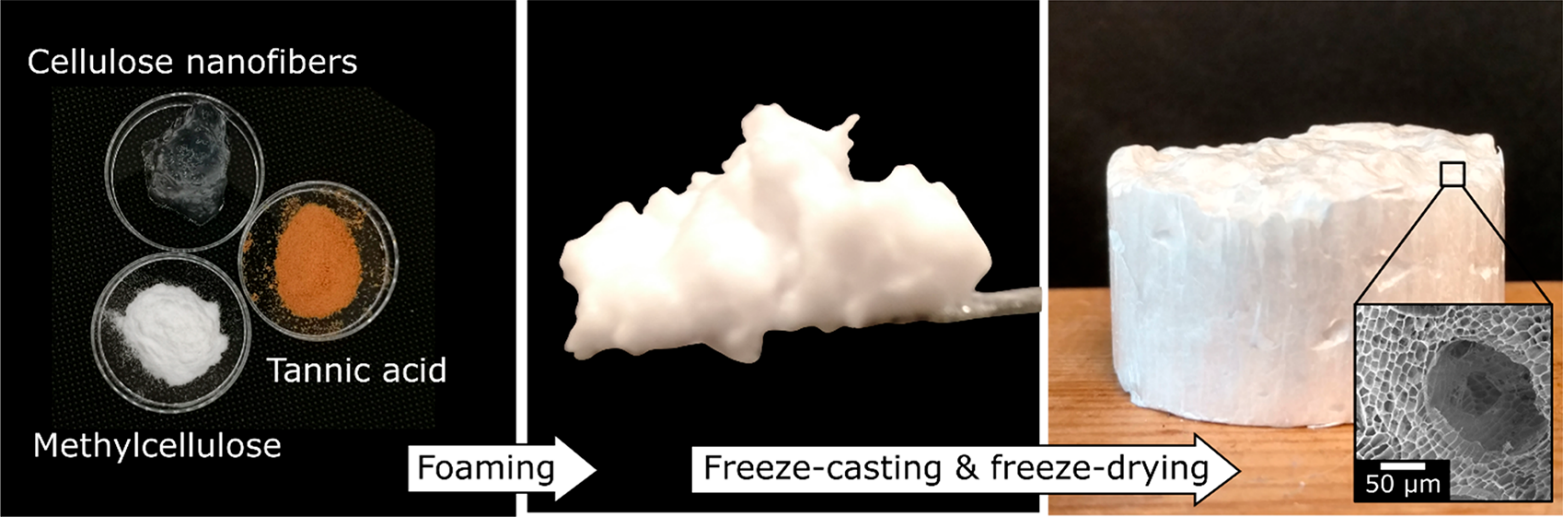

By forming and directionally freezing an aqueous foam containing cellulose nanofibrils, methylcellulose, and tannic acid, we produced a stiff and tough anisotropic solid foam with low radial thermal conductivity. Along the ice-templating direction, the foam was as stiff as nanocellulose–clay composites, despite being primarily methylcellulose by mass. The foam was also stiff perpendicular to the direction of ice growth, while maintaining λ_r_ < 25 mW m^–1^ K^–1^ for a relative humidity (RH) up to 65% and <30 mW m^–1^ K^–1^ at 80% RH. This work introduces the tandem use of two practical techniques, foam formation and directional freezing, to generate a low-density anisotropic material, and this strategy could be applied to other aqueous systems where foam formation is possible.

## Introduction

Nanocellulose-based foams and aerogels, in which large volumes of air are incorporated into scaffolds containing cellulose nanocrystals (CNC) or nanofibers (CNF), can have a thermal conductivity λ lower than that of air (26.2 mW m^–1^ K^–1^ at 295 K and 1 bar^1^).^2,3^ Anisotropic nanocellulose-based foams can have an especially low λ perpendicular to the aligned nanoparticles,^3−7^ which has been attributed to the lower λ perpendicular to the cellulose chain^8^ and to phonon scattering at the interfaces.^3−7^ Anisotropic foams are generated by directional ice templating, in which an aqueous suspension is frozen along a directional temperature gradient and then freeze-dried.^9^

Efforts to understand and lower the thermal conductivity of nanocellulose-based foams and aerogels suggest that phonon scattering at interfaces dominates;^3−7^ that is, that interfacial boundaries between nanoparticles greatly reduce thermal conductivity in nanocellulose assemblies.^8^ Thus, foams containing CNF with other nanostructured materials^4,10−12^ or even microfibrillated cellulose,^13^ can have lower thermal conductivity than their CNF-only analogues. Further, moisture causes swelling in nanocellulose-based materials, increasing the distance between nanofibrils and thus phonon scattering and limiting thermal conductivity in aligned nanocellulose foams at moderate relative humidity.^5^

We hypothesized that an aerogel that combined low density, hierarchical porosity, anisotropy, and multiple particle types could be obtained by directionally ice-templating and freeze-dying a wet nanocellulose-based foam; that is, a suspension containing large air-templated voids that are supported by nanocellulose and other particles. Aqueous suspensions of nanocellulose can be foamed after rendering the nanocellulose surface partially hydrophobic^14−16^ or including surfactant molecules.^17−22^ Isotropic dry foams have been generated by oven-drying wet cross-linked CNF-based foams,^17−19^ by freeze-drying CNC-based foams containing other polymers,^21,22^ and by freeze-drying wet foams composed of gelatin and silica precursors.^23^

In this study, we demonstrate how wet foams containing only the plant-based components CNF, methylcellulose, and tannic acid can be directionally frozen and dried to give anisotropic foams with a high mechanical strength and low thermal conductivity.

## Experimental Section

### Materials and Syntheses

#### Materials

Methylcellulose (MC, η = 8 × 10^3^ cPs at 2 wt % in H_2_O, 1.78 ± 0.14 methoxy groups per anhydroglucose unit) and tannic acid (94.0 wt %) were purchased from Alfa Aesar and used as received. A 0.87 ± 0.01 wt % suspension of TEMPO-oxidized cellulose nanofibrils (CNF) was prepared by oxidizing never-dried softwood sulphite pulp (Domsjö dissolving pulp, 3:2 spruce/pine, Sweden) with TEMPO (2,2,6,6-tetramethylpiperidin-1-yl)oxyl) according to the method of Saito et al.^24^ and disintegrating in a high-pressure microfluidizer.^25^ Conductometric titration^26^ indicated a surface carboxyl content of 1.10 mmol/g, and atomic force microscopic measurements showed an average CNF diameter of 3.0 ± 0.6 nm.^27^ Deionized water was used for all experiments.

#### Wet Foam Containing CNF, MC, and TA

The synthesis was based on published procedures.^18,21,22^ In the first step, a suspension of CNF and methylcellulose was prepared. Thus, a suspension of TEMPO-oxidized CNF (0.87 wt %, with the dry weight being labeled *m*_CNFdry_) was diluted with deionized water to a concentration of 0.55 wt % in a plastic jar. The mixture was stirred with a magnetic stir bar at room temperature while MC (3.7 × *m*_CNFdry_) was added. Once all MC was added, stirring was difficult, so the jar was manually swirled to wet and incorporate the MC into the suspension. The mixture was allowed to stir as well as possible overnight. In the second step, the suspension was homogenized and foamed on a rotor–stator mixer (Ultra Turrax T25, IKA) at 10000 rpm for 3 min. Then, with continued mixing, a solution of tannic acid (0.0875 × *m*_CNFdry_) in deionized water (48 × *m*_CNFdry_) was added dropwise. Once all of the solution had been added, a spatula was used to push the foam down in the jar, and the foam was subjected to the rotor–stator mixer for an additional 1 min. The wet foam had a total solid concentration of 2.0 wt % unless otherwise noted. Wet foams are labeled CNF–MC–TA.

#### AIT_CNF-MC-TA_

Custom-built molds were used for directional ice-templating. These consisted of a round Cu bottom plate attached to an empty Teflon cylinder. The wet CNF–MC–TA foam was viscoelastic, with a storage modulus of approximately 400 Pa at low shear, and was also shear-thinning (Figure S3), so it was most easily handled via extrusion. Thus, a spatula was used to load a 50 mL syringe with wet CNF–MC–TA foam, which was pressed into the mold in order to completely cover the Cu plate. Care was taken to minimize the occurrence of very large (several mm) bubbles or cavities in the viscous foam. The mold was filled to a height of 2–3 cm, then placed on a slab of CO_2_(s) and allowed to stand until the entire sample was frozen and ice crystals formed on top of the frozen foam. The Cu plate was removed from the bottom of the mold, and the frozen foam pressed out of the mold and into a refrigerated box. Some frozen foams were cut along or perpendicular to the direction of freezing in order to produce samples for imaging. The foams were quickly transferred to a freeze-dryer (Christ Alpha 1–2 LD plus) and evacuated at low pressure (∼0.025 mbar) for at least 48 h.

#### IT_CNF_

A suspension of CNF (0.87 wt %, dry weight of *m*_CNFdry_ g) was combined in a plastic beaker with deionized water (84 × *m*_CNFdry_ g). A rotor–stator mixer operating at 10000 rpm was used to homogenize the mixture, which was then centrifuged at 6000 rpm for 3 min to remove the bubbles formed during mixing. The suspension was poured into the custom mold described above and directionally ice-templated according to the method used for AIT_CNF-MC-TA_.

### Characterization Methods

Thermal conductivity was determined using a TPS 2500 S Hot Disk Thermal Analyzer (Hot Disk AB, Sweden) in the anisotropic mode. A Kapton 5501 sensor (6.4 mm radius) was sandwiched between a pair of identical foam cylinders (*d* = 3.7 ± 0.2 cm; height = 2.7 ± 0.2 cm) and thermal contact was guaranteed by placing a small weight (39 g) on top of the samples (contact pressure 398 ± 0.4 N m^2^). All measurements were performed with a heating power of 20 mW for 10 s. The samples were placed in a customized vessel that allowed the control of relative humidity (5–80%) and temperature (295 K).^28^ For each relative humidity (5, 20, 35, 50, 65, and 80%), five independent measurements were performed at intervals of 15 min, and at least two different pairs of identical foams were used. Further information on the determination of the thermal diffusivity and conductivity and the error analysis can be found in Supporting Information, S1.

Mechanical properties of the hierarchical foams were determined using compression testing at 295 K and 50% RH on an Instron 5966 universal testing machine (Instron, U.S.A.) that was equipped with a 100 N load cell. Specimens were conditioned at 295 K and 50% RH for at least 48 h before being weighed, measured with a digital caliper, and then compressed at a strain rate of 10% min^–1^. For compression along the direction of ice-templating, cylinders with *d* ∼ 1.9 cm and *h* ∼ 2 cm were measured as-synthesized. For compression perpendicular to the direction of ice-templating, a razor blade was used to cut a larger foam cylinder into a rectangular prism (ca. 1.3 × 1.3 × 1.9 cm) for measurement. The compressive Young’s modulus was determined from the slope of the initial linear elastic region of the stress–strain curve. The specific Young’s modulus was calculated by dividing the modulus by the apparent sample density.

Further characterization methods are described in the Supporting Information.

## Results and Discussion

**Figure 1:**
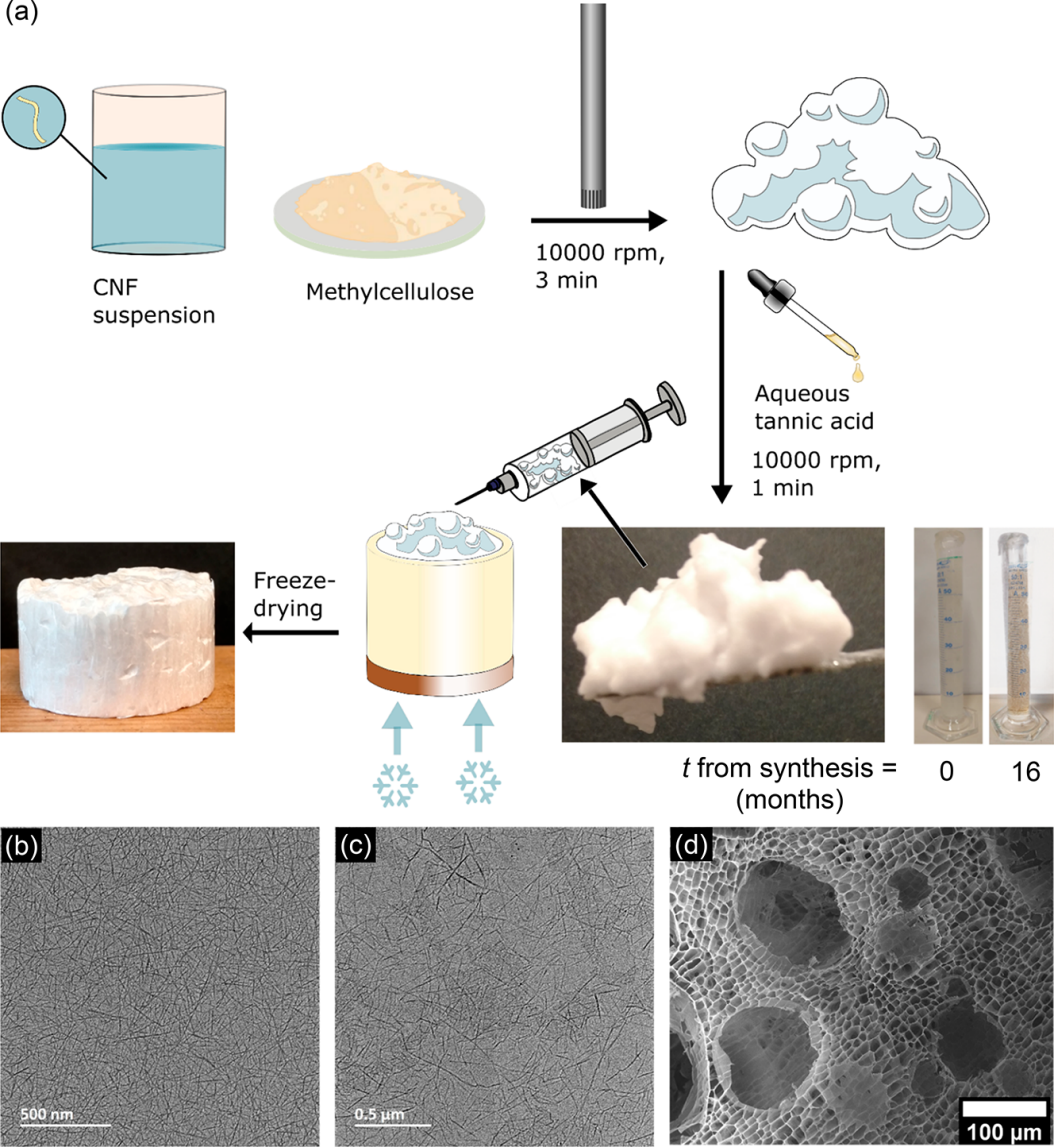
Preparation of AIT_CNF-MC-TA_. (a) Schematic illustration of the foam preparation, including photographs of the wet and dry foams containing cellulose nanofibers, methylcellulose, and tannic acid (*m*_CNF_/*m*_MC_/*m*_TA_ = 21:77:2, solid content in suspension = 2 wt %); (b) transmission electron microscope image of the CNFs; (c) transmission electron microscope image of a mixture of CNF and MC (*m*_CNF_/*m*_MC_ = 0.28); (d) scanning electron microscope image of AIT_CNF-MC-TA_.

The air- and ice-templated CNF-based foam was made by directionally ice-templating a wet foam (Figure 1a) that was supported by CNF (TEMPO-oxidized,^24^ TEMPO = 2,2,6,6-tetramethylpiperidine 1-oxyl), methylcellulose (MC), and tannic acid (TA), the latter of which supports smaller air bubbles in wet foams based on CNF and MC.^18,29^ The wet foam with *m*_CNF_/*m*_MC_/*m*_TA_ = 21:77:2 and a solids content of 2 wt % was produced using a two-step recipe based on those for related nanocellulose-based foams.^18,21,22^ First, an aqueous suspension of CNF and MC (Figure 1b,c) was foamed using high-shear mixing (Ultra Turrax T25), which then continued while a dilute aqueous solution of TA was added dropwise. Once all TA(aq) had been added, the mixture was foamed for one additional minute to give the wet foam (Figure 1a), which was extruded through a syringe into a custom-built mold composed of a Cu base and a detachable Teflon cylinder. The Cu plate was then placed on a block of CO_2_(s), and the foam was allowed to freeze from the bottom up before being removed from the mold and freeze-dried to give a solid foam (Figure 1a,d) that is labeled AIT_CNF-MC-TA_ for air- and ice-templated foam composed of CNF, MC, and TA.

### Preparation and Stability of Wet Foams

To date, anisotropic thermal conductivity has been studied as a function of humidity primarily for solid foams containing only nanocellulose (CNC or CNF),^5,7^ making it possible to rationalize the results based on an understanding of the constituent particles. However, the dry foam synthesized in this work contained not only CNF, but also the MC and TA used to support stable wet foam,^18^ and we therefore investigated the nature of the particles in dilute suspensions of CNF with methylcellulose and tannic acid. Measurements of interfacial tension, ζ-potential, and TEM produced no evidence that MC interacted strongly with the CNFs in dilute aqueous suspension (see Supporting Information and Figures 1b,c and S1), contrary to the case of methylcellulose and sulfonated CNCs,^30−32^ but similar to the case of methylcellulose with mechanically fibrillated cellulose.^33,34^ Thus, we expect the MC and CNF to exist separately in suspension.

Upon foaming, MC and CNF stabilize air–water interfaces in different ways. MC is surface-active; we found that a suspension of 0.005 wt % MC in H_2_O had an interfacial tension of 52.6 ± 0.1 mN m^–1^ against air at room temperature (cf. 72 mN m^–1^ for pure water^35^), and fluorescence microscopy has shown MC concentrated at the air–water interface in foams supported by MC and cellulose nanocrystals.^22^ TEMPO-oxidized CNFs such as those used here are hydrophilic but can stabilize emulsions by adsorbing to droplets (Pickering emulsion) and via network formation,^36,37^ and fluorescence microscopy has shown CNF assembled at the air–water interface in foams supported by MC, CNF, and montmorillonite clay.^18^ Here, a suspension of 0.005 wt % MC and 0.014 wt % CNF had an interfacial tension of 40.0 ± 0.1 mN m^–1^ against air; thus MC and CNF stabilized the air–water interface more than MC alone. Therefore, upon foaming the suspension of MC and CNF, we expect both types of particles to be organized at the air–water interface,^18,22^ but without strongly interacting with each other, and for a CNF network to form in the suspension phase.^15^

In the next step, high-shear mixing continued while a dilute aqueous solution of TA was added dropwise in order to avoid the formation of colloidal MC–TA complexes away from the air–water interface.^18,38^ When TA was included in dilute aqueous suspensions with CNF and MC (see Supporting Information), it protonated some carboxyl groups on the CNF and interacted with MC. In the case of our wet foams, however, TA was added to a preformed foam supported by MC and CNF, which will influence its interactions with these biopolymers. TA improves the stability of foams supported by MC alone.^29^ Further, in wet CNF-based foams containing MC and montmorillonite clay, TA addition produced smaller bubbles and raised the rheological storage modulus of the foam.^18^ On the other hand, adding TA to CNF–MC foams with *m*_MC_/*m*_CNF_ = 3.7 did not give statistically significant changes in bubble size (average bubble size was 80 ± 30 μm after TA addition; see Figure S2) or rheological storage modulus (Figure S3). Thus, the impact of TA on the wet CNF–MC–TA foams was small. MC, on the other hand, was critical to foam formation, as subjecting a suspension of CNF and TA to high-shear mixing yielded a gel with few incorporated air bubbles. Notably, the wet CNF–MC–TA foams produced here were very stable; when a column of foam containing as little as 0.9 wt % total solids was protected from evaporation using Parafilm, it only lost approximately 3–4% of its height over 16 months (Figure 1).

### Directional Freezing of Wet Foams and Alignment of Particles

**Figure 2:**
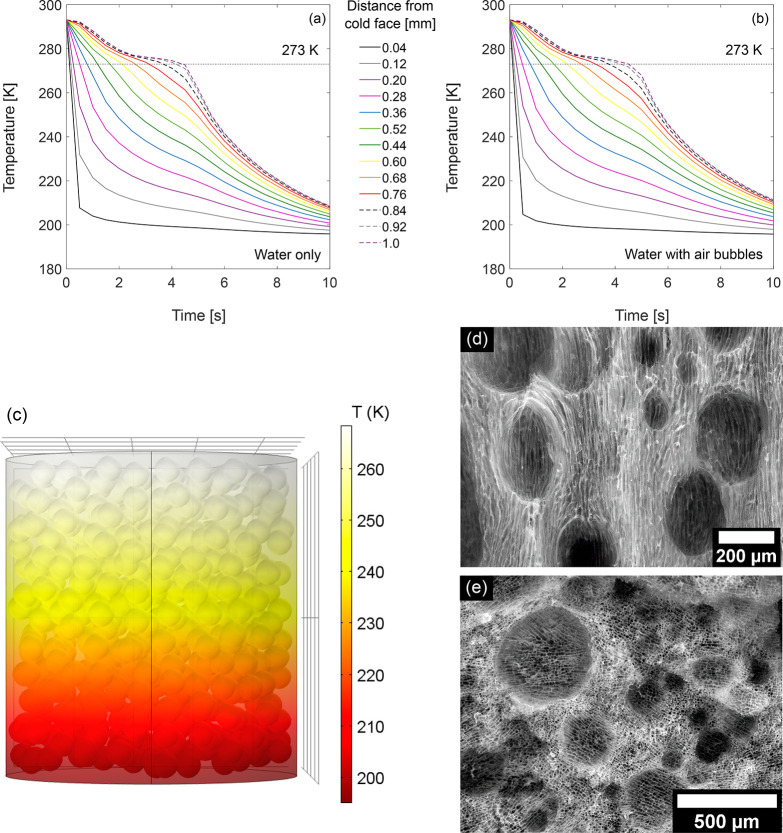
Directional freezing of aqueous foams in a cylinder with one cold face. (a, b) Calculated temperature as a function of simulation time and distance from the cold (195 K) face of a cylinder (*r* = 0.46 mm, *h* = 1 mm) containing (a) water and (b) water and 500 randomly placed air bubbles with *d* = 80 μm. (c) Temperature distribution in the bubble-containing cylinder after simulating 5 s of time. (d, e) SEM images of the air- and ice-templated foam AIT_CNF-MC-TA_. Following directional freezing, the sample was cut parallel (d) or perpendicular (e) to the freezing direction and then freeze-dried.

A multiphysics simulation indicated that randomly arranged air bubbles with *d* = 80 μm had only a minor effect on heat flow in a cylinder of water that had a constant temperature of 195 K at one face (Figures 2a,b and S4). The cooling front moved slightly more slowly in a cylinder containing air bubbles compared to a cylinder of pure water, as is expected given that air is a poor conductor of heat compared to water and ice. However, in both cases, cooling occurred perpendicular to the cold face, and the bubbles caused only local (less than the radius of the bubble) variations in temperature parallel to the cold face (Figures 2c and S4). Thus, despite the presence of small, spheroid air bubbles, freezing was highly directional, and we therefore expected that wet CNF–MC–TA foam could be directionally ice-templated.

### Structure and Alignment of Solid Foams

The air- and ice-templated solid foam AIT_CNF-MC-TA_ had an apparent density ρ_app_ = 18 kg m^–3^ (Table 1), and displayed two types of macropores (Figures 2d,e and S5): the columnar ice-templated pores (average size 14 ± 4 μm) and spheroid pores templated around the air bubbles of the wet foam. The spheroid voids were, on average, larger (160 ± 80 μm; Figure S6) than the air bubbles observed in the wet foam (80 ± 30 μm, Figure S2), likely due to coarsening of the foam prior to and during freezing. The spheroid voids in air- and ice-templated AIT_CNF-MC-TA_ (Figure 2d,e) were bordered by sometimes-incomplete curved interfaces (Figure S5) that were formed from the particles that had supported the air bubbles in the wet foam. The alignment of the solid, as assessed from the SEM images (Table 1), in AIT_CNF-MC-TA_ (*f* = 0.84) was slightly lower than that of an ice-templated foam containing only CNF, which here is labeled IT_CNF_ for ice-templated foam composed of CNF (see SEM images, Figure S7) and had *f* = 0.90. This relatively small difference in alignment was supported by estimates of the relative amount of the solid material in the walls of the ice-templated versus air-templated voids, which was based on the average wall thickness and density of the foam, as well as the net apparent density of the foam (see calculation in the Supporting Information), and suggested that >90% of the mass of AIT_CNF-MC-TA_ existed in the walls of ice-templated rather than air-templated voids. Thus, although the presence of curved solid regions in AIT_CNF-MC-TA_ may decrease the anisotropy of its properties, these regions contained a minority of the solid in the foam.

**Table 1:** Chemical Composition and Physical Properties of the Foams

						nanopores^c^		orientation^e^ [−]
	solids in wet foam^a^ (wt %)	ρ_app_^b^ (kg m^–3^)	*S*_BET_^c^ (m^2^ g^–1^)	pore wall density^d^ (g cm^–3^)	porosity^d^ [−]	volume (cm^3^ g^–1^)	avg size (nm)	*f*, SEM
IT_CNF_^f^	0.5	6.5 ± 0.1	11.9	1.53	0.999	0.028	10.3	0.90
AIT_CNF-MC-TA_	2.0	18.0 ± 0.5	13.2	1.38	0.987	0.024	8.6	0.84

a Total content of cellulose nanofiber (CNF), methylcellulose (MC), and tannic acid (TA) in the wet foam (for AIT_CNF-MC-TA_) or suspension (for IT_CNF_) prior to ice-templating. For CNF–MC–TA, *m*_CNF_/*m*_MC_/*m*_TA_ = 21:77:2.

b Apparent density, calculated from the dimensions and mass of samples equilibrated at 295 K and 50% relative humidity.

c From the adsorption branch of the N_2_ adsorption isotherm, see Figure S8; *S*_BET_ = Brunauer–Emmett–Teller^39^ surface area, calculated over approximately *P*/*P*_0_ = 0.05–0.30.

d See Supporting Information for details of calculations.

e Structural orientation from SEM; see Supporting Information for details.

f We have previously reported the data for IT_CNF_ (except for orientation parameter).^27^

### Thermal Conductivity and Moisture Uptake

**Figure 3:**
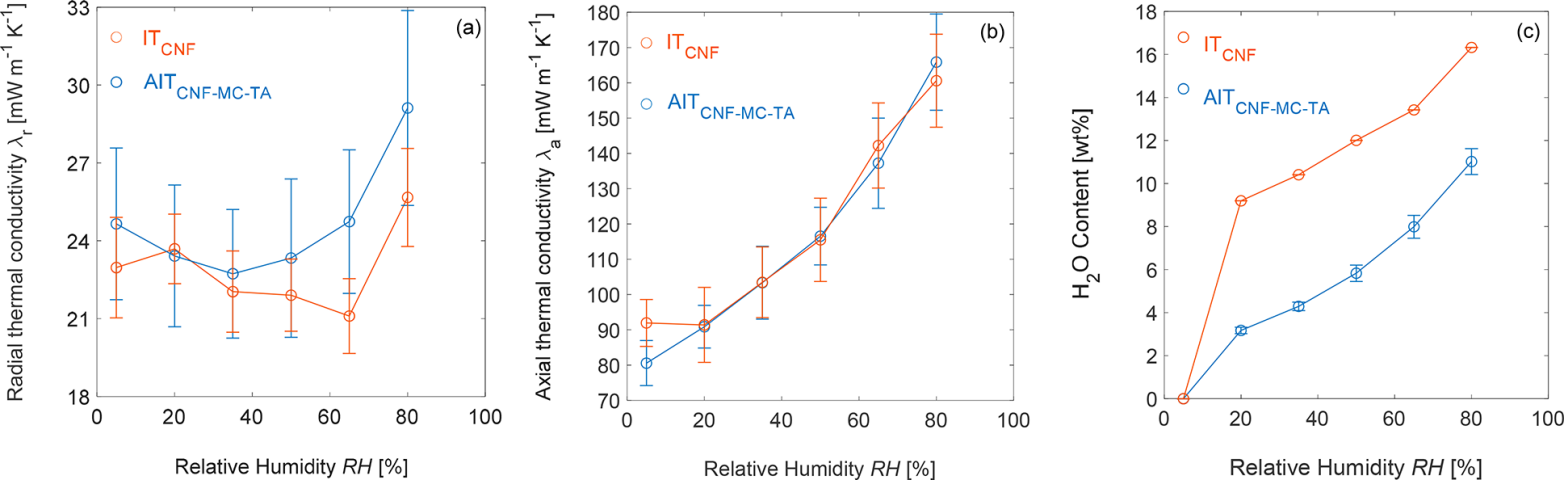
(a) Radial thermal conductivity λ_r_, (b) axial thermal conductivity λ_a_, and (c) moisture uptake of the IT_CNF_ and AIT_CNF-MC-TA_ foams as functions of relative humidity. In (a) and (b), the error bars represent the relative uncertainty of the thermal conductivity from each set of measurements at the relevant relative humidity (details in the Supporting Information); in (c), the error bars represent the standard deviation from three independent measurements.

The thermal conductivity in the radial direction (λ_r_) of the AIT_CNF-MC-TA_ foam was 25 mW m^–1^ K^–1^ under dry conditions (5% RH and 295 K). It remained at a value close to or slightly below the value for air at intermediate RH, then rose to 30 mW m^–1^ K^–1^ at 80% RH (Figure 3a). Thus the λ_r_ of AIT_CNF-MC-TA_ under RH ≤ 50% was similar to that of IT_CNF_ (Figure 3a), despite the significantly higher density of AIT_CNF-MC-TA_ (Table 1), and both foams displayed a value for λ_r_ below the thermal conductivity of dry air for RH ≤ 65%. The axial thermal conductivities of AIT_CNF-MC-TA_ and IT_CNF_ were very similar (Figure 3b). Despite their similar thermal conductivities, the air-templated AIT_CNF-MC-TA_ foam took up less water than IT_CNF_ (Figure 3c). The higher porosity of IT_CNF_ (Table 1) may have endowed it with more H_2_O-accessible sites for adsorption.

The very low radial thermal conductivity of IT_CNF_ at intermediate RH corresponds well to previous studies on ice-templated CNF foams at intermediate RH.^7,13,40,41^ In fact, the λ_r_ of AIT_CNF-MC-TA_ showed a similar dependence on RH that was observed for IT_CNF_^27^ and other ice-templated CNF foams;^5^ that is, λ_r_ first decreased, then increased, with RH. This behavior has been attributed to two competing effects: the humidity-induced swelling of the fibrillar foam walls causes the thermal boundary conductance between particle surfaces to decrease as the distance between them increases; whereas at higher humidity, the replacement of air by water in the interparticle spaces increases the thermal boundary conductance.^5^ Nevertheless, even at 80% RH, the λ_r_ of AIT_CNF-MC-TA_ was below the thermal conductivity of typical polystyrene foams (30–40 mW m^–1^ K^–1^).^42^ The λ_r_ of AIT_CNF-MC-TA_ was slightly lower than that of an analogous foam prepared with only 60% as much TA (AIT_CNF-MC-LTA_; Figure S9) for all values of RH, which suggests that addition of TA did not increase λ_r_.

### Mechanical Properties

**Figure 4:**
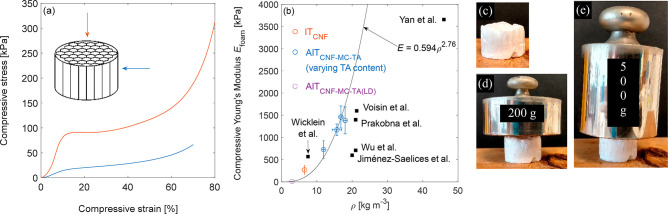
Compressive properties of AIT_CNF-MC-TA_ and IT_CNF_ at 295 K and 50% RH. (a) Representative stress–strain curves for the compression of AIT_CNF-MC-TA_ along and perpendicular to the direction of ice templating. (b) Compressive Young’s modulus as a function of density for AIT_CNF-MC-TA_, IT_CNF_, and some reported foams.^4,18,44−47^ The data point for IT_CNF_ has been reported.^27^ (c–e) Digital photographs of an AIT_CNF-MC-TA_ foam weighing 72.3 mg, without and with weights.

The anisotropic AIT_CNF-MC-TA_ foam was noticeably stiff, even perpendicular to the direction of ice growth. When compressed along the direction of ice growth (Figure 4a), the foam displayed a stress–strain curve typical of elastic–plastic cellular foams;^43^ stress increased nearly linearly at first, reaching a maximum and then a plateau before increasing sharply during densification at higher strain. AIT_CNF-MC-TA_ had a specific compression Young’s modulus of 80 ± 20 kNm kg^–1^ (Figure 4b) along the direction of ice growth. Thus, despite being composed of plant-based organics, AIT_CNF-MC-TA_ was as stiff per unit density as the stiffest CNF-based foams reported, including cross-linked (80 kNm kg^–1^)^44^ or composite (77 kNm kg^–1^)^4^ ice-templated foams and an isotropic cross-linked composite foam (74 kNm kg^–1^),^18^ as well as a holocellulose honeycomb (67 kNm kg^–1^).^45^ AIT_CNF-MC-TA_ was stiffer than an isotropic CNF-only foam that was templated around emulsion droplets (up to ∼50 kNm kg^–1^; ∼30 kNm kg^–1^ for a foam with a similar density to AIT_CNF-MC-TA_)^46^ as well as a physically and chemically cross-linked CNF foam (∼34 kNm kg^–1^).^47^ Varying the content of TA in AIT_CNF-MC-TA_ over 0–3.6 wt % had no clear effect on compression stiffness; rather, the specific stiffness and toughness of these foams were similar considering the variation in experimental data (Table S1). Thus TA could be omitted with little effect on the specific mechanical properties of the solid foam. The density of the AIT_CNF-MC-TA_ foams varied with TA content, and a low-density analogue of AIT_CNF-MC-TA_, here labeled AIT_CNF-MC-TA(LD)_ and having ρ_app_ = 3.0 ± 0.1 kg m^–3^, was generated for comparison. The compressive Young’s modulus of cellular solids can be related to their density by the power law in eq 1, where *E* is Young’s modulus, the subscripts *cs* and *s* indicate the cellular solid (foam or aerogel) and the constituent solid, respectively, and *a* is an exponent that depends on the type of cellular solid.

For a theoretical honeycomb structure, *a* = 1,^48^ and this relationship has been confirmed for ice-templated holocellulose honeycombs.^45^ For an isotropic open-celled solid, *a* = 2,^43^ and nanocellulose foams fitting this model have also been reported.^49−51^ However, cellulose^52^ and nanocellulose^40,46,52^ foams and aerogels can also have *a* > 3 when increases in their density reflect increases in network connectivity.^46^ The structure of the AIT_CNF-MC-TA_ foams is complicated in that it contains both the elongated (honeycomb-like) ice-templated macropores and the isotropic (cellular) spheroid macropores. Further, as expected for frozen and freeze-dried isotropic nanocellulose foams,^49,51^ the isotropic macropores are bordered by sheets rather than struts (i.e., they are partially closed cellular solids). Nevertheless, assuming that solid density and solid Young’s modulus are constant despite small changes in the TA content of the foams, the Young’s modulus of the air- and ice-templated foams reported here could be fit to eq 1, with *a* = 2.75 (Figure 4b).
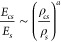
1

AIT_CNF-MC-TA_ was stiff even perpendicular to the direction of ice crystal growth, with a specific Young’s modulus of 10 ± 2 kNm kg^–1^. This was higher than that reported for a CNF-only foam (2–3 kNm kg^–1^),^47^ though lower than a physically and chemically cross-linked foam (21 kNm kg^–1^),^47^ and the strongest of three ice-templated clay–CNF composites (31 kNm kg^–1^).^53^ The compression stress–strain curves for AIT_CNF-MC-TA_ along and perpendicular to the direction of ice growth (Figure 4a) were qualitatively similar to those reported for a rigid anisotropic open polyurethane foam,^54^ in that both the Young’s modulus and the plastic plateau were higher along the direction of ice growth. In the anisotropic polyurethane foam, the anisotropy of the mechanical properties depended on the pore dimension in the material. However, in CNF foams, the pore walls themselves are composed of highly anisotropic particles. Nyström and co-workers found that the ratio of Young’s moduli along and perpendicular to the direction of ice growth was 11–12 for ice-templated CNF foams; this ratio fell to 5–7 upon physical or chemical cross-linking, and was <2 with both types of cross-linking.^47^ The Young’s modulus of AIT_CNF-MC-TA_ was 8× higher along than perpendicular to the direction of ice growth, so it was less anisotropic than a CNF-only foam, but more isotropic than a cross-linked foam. This lower anisotropy in AIT_CNF-MC-TA_ than in a CNF-only foam is likely due to a combination of the curved regions of solid as well as to the inclusion of methylcellulose.

## Summary and Conclusions

We combined two operationally simple procedures, foaming and ice-templating, to generate a stiff and tough anisotropic plant-based foam with low radial thermal conductivity. An aqueous foam containing cellulose nanofibrils, methylcellulose, and tannic acid was prepared using a high-shear mixer and directionally ice-templated to give the solid foam AIT_CNF-MC-TA_, which contained columnar and spheroid micrometer-sized pores. By mass, AIT_CNF-MC-TA_ was almost 70% methylcellulose, a cheap plant-based polymer; however it is among the stiffest reported nanocellulose-based foams to date, reaching similar specific compression Young’s modulus as nanocellulose–clay composites. AIT_CNF-MC-TA_ also combined a high stiffness in the direction perpendicular to the direction of ice growth with a low λ_r_ for RH up to 50%. Even at 80% RH, the λ_r_ for AIT_CNF-MC-TA_ was less than 4 mW m^–1^ K^–1^ higher than that of the CNF-only foam, and lower than the thermal conductivity of typical polystyrene foams (30–40 mW m^–1^ K^–1^).^42^

AIT_CNF-MC-TA_ was synthesized by generating and directionally freezing wet foams. There are multiple routes to aqueous foams based on cellulose nanomaterials (CNC and CNF), including wet foams that also contain synthetic polymers and inorganics, and we therefore expect the procedure described here to provide a route to a wide variety of functional anisotropic materials.

## Supporting Information Available

Complete details of foam characterization, supplementary results, and discussions regarding the interactions of CNF, MC, and TA in dilute aqueous suspension (PDF)
