# Supplementary material for: A Stiff, Tough, and Thermally Insulating Air- and Ice-Templated Plant-Based Foam
Source: Biomacromolecules. 2022 May 27;23(6):2595–602. doi: 10.1021/acs.biomac.2c00313 (PMC9198970; doi:10.1021/acs.biomac.2c00313)
Supplement: Supplementary file 1 — bm2c00313_si_001.pdf [file bm2c00313_si_001.pdf]

Supporting Information for:

A stiff, tough, and thermally insulating air- and ice-templated plant-based foam

Tamara L. Church,[a] Konstantin Kriechbaum,[a] Carina Schiele,[#,a] Varvara Apostolopoulou-Kalkavoura,[#,a] Seyed Ehsan Hadi, [a,b] and Lennart Bergström*,[a]

[a] *Department of Materials and Environmental Chemistry, Stockholm University, Stockholm, 10691 Sweden*

[b] *Wallenberg Wood Science Center, Department of Materials and Environmental Chemistry, Stockholm University, Stockholm, 10691, Sweden*

# These authors contributed equally

* To whom correspondence should be addressed

Item **Page**

S1. Characterization methods S2

S2. Interactions of CNF, MC, and TA in dilute aqueous suspension (including Figure S1) S7

S3. Additional data S7

Figure S2: Bubble sizes in wet CNF–MC and CNF–MC–TA foams S7

Figure S3: Rheology of CNF–MC and CNF–MC–TA foams S7

Figure S4: Modelled heat transfer in water or foam S8

Figure S5: High-magnification SEM images of **AIT$_{CNF-MC-TA}$** S9

Figure S6: Distribution of spheroid bubbles in **AIT$_{CNF-MC-TA}$** S9

Figure S7: SEM images of **IT$_{CNF}$** S9

Figure S8: N_2 sorption isotherms for the solid foams S10

Figure S9: Thermal conductivities of foams with different tannic acid contents S10

Table S1: Properties of air- and ice-templated foams with varying tannic acid contents S10

S4. References S11

S1. Characterization Methods

Contact Angle Measurements Aqueous suspensions with the desired concentrations of cellulose nanofibers (CNF), methylcellulose (MC), and tannic acid (TA) were prepared from stock solutions, with TA always being added last. The mixtures were stirred for several hours using magnetic stir bars, then allowed to stand until they no longer bore a foam phase (hours to overnight, depending upon the concentration). An aliquot of suspension was drawn into a 1-mL syringe. A flat-ended needle of known thickness (measured with a caliper) was affixed to the syringe, which was mounted in the sample dispenser of a DSA25E drop shape analyzer (Krüss). Separately, n-dodecane was placed in a quartz cuvette (1 × 1 mm). The stage and dispenser heights were adjusted so that the needle end was submerged in n-dodecane and both were visible in the instrument camera. The image was focused and the scale calibrated to the needle width. A sample drop was expressed; drops were as large as possible without falling from the syringe (5–15 µm, depending on the solids in

suspension). The drop size and shape was monitored by the instrument for 15–20 min, with measurements bring recorded every 20 s. The interfacial tension for a given experiment was taken as the average ± standard deviation of the values from the final 3 min of measurement. Any measurement in which the drop shape factor was below 0.40 or above 0.50 was discarded. The n-dodecane was refreshed between samples. Measurements were performed at room temperature, which varied from 293 to 295 K.

Zeta potential (ζ-potential) measurements Samples for ζ-potential measurements were prepared using the same procedure as those for contact angle measurements. Measurements were performed on a Zetasizer Nano ZS at 298 K using disposable folded capillary cells.

Transmission electron microscopy Transmission electron microscopy (TEM) on a JEM-2100F microscope was used to record images of dispersions deposited onto a carbon-coated copper TEM grid at a 200 kV accelerating voltage. The images were processed with Gatan Inc. software.

Air in wet foam A small sample of wet foam (3.9 g) was prepared and extracted through a syringe into a shallow plastic cylinder. Its density was calculated from its mass and dimensions to be 420 kg m^{-3}. The sample was frozen isotropically to obtain a solid. The following day, the solid was transferred to a centrifuge vial and weighed. It was then allowed to thaw and centrifuged to yield a clear suspension, and the volume was evaluated using the markings on the centrifuge tube. The suspension density was 1010 kg m^{-3}. Then, using by assuming that the density of the suspension phase is not significantly changed by foaming, and given that $\rho_{air} << \rho_{suspension}$, we can estimate that the wet foam was 42 vol% suspension, and 58 vol% air. The air bubbles were measured from images of foam on a microscope slide taken using a DinoCapture 2.0 microscope. The measurements were made in the Fiji[1] distribution of ImageJ[2] and only bubbles in the focal plane were measured. The Feret diameter and Feret minimum diameter were averaged to give the diameter of the bubble, and 80 bubbles were measured for each sample (i.e. foam before and after TA addition).

Heat transfer in wet foam Heat transfer in the wet foam was modelled using COMSOL Multiphysics 5.6 software.[3] The model was composed of 500 spheres, each 80 µm in diameter (the average diameter of bubbles in the wet foam, see above). These were randomly distributed in a cylinder with radius 0.46 mm and height 1 mm by using Matlab 2020a to generate random, non-overlapping spheres.[4] Thus the bubbles used in the computation occupied 20% of the cylinder volume, which is less than the estimated (see above) 58% of the volume of the wet foam that was air. However, generating random non-overlapping spheres to occupy a high volume fraction in a fixed space is computationally expensive, and we therefore chose to work with a smaller volume fraction. The array of spheres was converted to a .dxf file using DXFLib,[5] then to an .stl file using 3D-Convert.[6] The cylinder was created in COMSOL. The cylinder material was approximated using water as a phase-change fluid and the spheres were air. One flat face of the cylinder was set at a constant temperature of 195 K to represent the copper plate in contact with dry ice, and the other was open boundary. The curved surfaces of the cylinder were set as thermally insulated in order to represent the Teflon cylinder. The initial temperature was 295 K for all materials and surfaces except the flat face at 195 K. The temperature profile of

the model was calculated at 0.5-s intervals for 10 s. For comparison, a separate model was built in which the spheres were not present. All of other parameters were retained.

Scanning electron microscopy Most SEM images were acquired on a Hitachi TM3000 electron microscope in charge-up reduction mode at an acceleration voltage of 15 kV in Analysis Mode (higher emission current). For higher-magnification images (Figure S5), samples were coated with Au nanoparticles and imaged on a JEOL JSM-7000F electron microscope using an accelerating voltage 15 kV and in secondary electron mode. Images were processed in the Fiji[1] distribution of ImageJ.[2] The orientation of the foam walls were evaluated by applying the ImageJ plugin OrientationJ[7,8] to images taken at 50× to produce a histogram of orientations. The local tensor was taken to be three pixels. For $\mathbf{AIT_{CNF\text{–}MC\text{–}TA}}$, the orientation was evaluated for three separate images at three different positions on the foam; only one image was analysed for $\mathbf{IT_{CNF}}$. The orientation was evaluated by fitting the orientation distribution to a single Gaussian function. The full width at half maximum (FWHM) of the fitted function was used to derive the orientation function according to Eq. S1.

$$f = \frac{180 - FWHM}{180} \tag{S1}$$

Textural properties The apparent density ρ_{app} of a foam was calculated from the masses and volumes of samples that had been equilibrated for a minimum of 24 h at 295 K and 50% RH. The volumes of the cylinders were calculated from the diameter and height, each taken as an average of two or three caliper measurements. Apparent densities are the average of at least five samples.

N_2 sorption isotherms were measured at 77 K on a Micromeritics ASAP 2020 sorption analyzer. Samples were manually compressed perpendicular to the direction of ice growth and degassed at 343 K for 14 h before measurement. The Brunauer–Emmett–Teller surface area[9] was calculated over a relative pressure range of approximately P/P_0 = 0.05–0.3, with the exact limits chosen to give the best correlation coefficient for $1/(Q(P/P_0–1))$ (where Q is the amount of N_2 adsorbed) vs. P/P_0 over a minimum of five data points. The volume of pores up to 300 nm in diameter, here called the nanopore volume V_{np}, was calculated from the adsorption branch of the isotherm using the Barrett–Joyner–Halenda method.[10] As the samples had been compressed prior to measurement, V_{np} is taken to represent only pores in, and not between, the foam walls.

The pore wall density was calculated from the skeletal density ρ_{skel} (taken as a weighted average of the densities of cellulose, methylcellulose, and tannic acid) and nanopore volume of a sample via Eq. S3.

$$Pore\ wall\ density = \frac{1}{V_p + \frac{1}{\rho_{skel}}} \tag{S3}$$

The percent porosity of a sample was calculated using Eq. S4.

$$Porosity = 100 * \left(1 - \frac{\rho_{app}}{\rho_{skel}}\right) \tag{S4}$$

The wall thickness and macropore sizes were evaluated from SEM images of the foams that had been cut perpendicular to the freezing direction (i.e. radial cuts). The wall thickness was measured from high-magnification images and is given as the average ± standard deviation of a total of at least ten measurements from two different images. Columnar macropores were also measured from high-resolution SEM images. A polygon was drawn on the edges of the pore and the largest (Feret) and smallest (min Feret) caliper measurements of the polygon were averaged to give the size of the pore. The reported columnar pore sizes give the average ± standard deviation of a minimum of 50 pores for each foam. For $\mathbf{AIT_{CNF\text{-}MC\text{-}TA}}$, the spheroid voids were fit to ellipses, and the Feret diameter of the ellipse was taken as its size. The size distributions and average ± standard deviation represent 49 spheroid pores.

Estimated distributions of air and solid in $\boldsymbol{AIT_{CNF\text{-}MC\text{-}TA}}$ The fractions of spheroid and columnar micrometer-sized pores, as well as the fraction of solid surrounding each of these, were estimated assuming that the pore wall thickness and pore wall density of the foam were uniform, and that all pores were average. Thus the following estimate was calculated for a pore wall thickness d_{wall} = 90 nm, pore wall density ρ_{wall} = 1340 kg m^{-3}, rectangular columnar pores with $l \times w$ = 18 × 10 µm, and spheroid pores with d_{sph} = 160 µm. As the SEM images were measured under vacuum, the dry density of $\mathbf{AIT_{CNF\text{-}MC\text{-}TA}}$, ρ_{dry} = 16.4 kg m^{-3}, was used for the estimate. We assume that all solid is part of a wall that borders either a sphere or a rectangle. In order to avoid double counting of the walls of face-sharing rectangles, a rectangular pore was considered to have one short and one long wall. Let x = number of centimeters of rectangular pore, and y = number of spherical pores. Then the total volume of micrometer-sized pores V_{mic} in cm^3, is given by Eq. S5, and the mass of the solid is given by Eq. S6.

$$V_{mic} = \big(x(l - d_{wall})(w - d_{wall})\big) + \frac{4\pi y}{3}\left(\frac{d_{sph}}{2} - d_{wall}\right)^3 \tag{S5}$$

$$m = x(l + w)(d_{wall})(\rho_{wall}) + \frac{4\pi y \rho_{wall}}{3}\left\{\left(\frac{d_{sph}}{2}\right)^3 - \left(\frac{d_{sph}}{2} - d_{wall}\right)^3\right\} \tag{S6}$$

Substituting and solving for x and y, we find that 1 cm^3 of dry $\mathbf{AIT_{CNF\text{-}MC\text{-}TA}}$ contains approximately 4.6 × 10^5 cm of columnar pores and 8.6 × 10^4 spherical pores. Thus the ~83% of the pore volume is contained in columnar pores, and ~95% of the solid is present as the wall of a columnar pore.

H_2O Uptake Foam cylinders (20–40 mm in diameter) were placed in a tared weighing dish on a Sartorius BSA124S analytical balance, which was contained within a Climacell EVO temperature- and humidity-controlled chamber. The balance was connected to a computer located outside the chamber, which recorded the sample mass was recorded every 5 min. For each sample condition, the heat and humidity were altered over a period of 2 h, then held constant for 4 h. The average and standard deviation of the masses recorded in the final hour of each condition are reported. The measured conditions were: 313 K at 20% RH (which approximates the "dry" condition at 295 K), then 295 K at 20, 35, 50, 65, and 80% RH. The moisture uptake was used to calculate Cp_{wet} and the wet density (see below).

Thermal conductivity The radial thermal diffusivity and conductivity were determined using the Cp_{wet} and the wet density of the foams as input. The axial thermal diffusivity and conductivity were obtained using the software provided by the Hot Disk as previously described.[11] The volumetric shrinkage of the foams at different RH was estimated by measuring their volume after each moisture cycle, and the sample density was corrected using the measured volume. The error bars represent the relative uncertainty of the thermal conductivity from each set of measurements at the relevant relative humidity. This was estimated, as previously reported,[12] by the error propagation analysis of the uncertainties of the thermal diffusivity (α), the density (ρ) and the specific heat capacity (Cp_{wet}) using Eq. S7.

$$\Delta\lambda = \lambda \sqrt{\left(\frac{\Delta\alpha}{\alpha}\right)^2 + \left(\frac{\Delta\rho}{\rho}\right)^2 + \left(\frac{\Delta Cp_{wet}}{Cp_{wet}}\right)^2} \tag{S7}$$

The random uncertainties of α, ρ and Cp_{wet} were based on estimates of the average standard deviations (SD) obtained from repeated measurements of several samples (at least 10 per sample for α, at least 4 per sample for ρ and 5 in total for Cp_{wet}) which were multiplied with 1.65, relating to a confidence interval of 95%. The systematic uncertainty of α was estimated to be 5%,[13] while no systematic uncertainty was considered for ρ and Cp_{wet}. ΔCp_{wet} incorporates the water uptake at different relative humidity which was measured separately in a humidity chamber.

Differential scanning calorimetry The specific heat capacity at constant pressure (Cp) of the samples was determined using a DSC 214 Polyma (Netzsch, Germany). The samples were dried in an oven at 105 °C before and after pressing them into a DSC pan which was closed with a lid and pierced with a needle. The DSC measurements were performed by measuring first an empty reference sample, and afterwards a sapphire sample used as standard sample and the oven-dried sample. All three types of samples were first heated to 105 °C for 10 min before the Cp_{dry} was measured between −20 and 50 °C at a heating rate of 10 °C min^{-1} under a N_2 atmosphere. The average Cp_{dry} at 22 °C of five independent measurements was considered for the calculations.

The wet specific heat capacity (Cp_{wet}) was calculated according to Eq. S8.

$$Cp_{wet} = (1 - H_2O_w) \times Cp_{dry} + Cp_{H_2O} \times H_2O_w \tag{S8}$$

where the Cp_{H2O} is the Cp of water obtained from the literature and H_2O_w is the moisture uptake by mass calculated during the humidity chamber experiments described above.

S2. Interactions of CNF, MC, and TA in dilute aqueous suspension

In order to understand the composition of the dry foam, we investigated the nature of the particles in dilute suspensions of CNF with methylcellulose and tannic acid. Although investigations into suspensions containing CNC and MC have indicated that MC chains wrap around CNC rods,[14–16] there are few investigations of CNF–MC systems, and they use mechanically fibrillated cellulose.[17,18] Contrary to the case with sulfonated CNCs,[14–16] but similar to the case with mechanically fibrillated cellulose,[17,18] the MC did not appear to associate strongly with the CNFs used in this study, which were TEMPO-oxidized (TEMPO = 2,2,6,6-tetramethylpiperidine 1-

oxyl)[19] and thus bore sodium carboxylate functional groups on their surfaces. A suspension of 0.013 wt% CNF in deionized water displayed an interfacial tension of 29.7 mN m^{-1} against dodecane (Figure S1). Oxidized CNFs such as those used here are hydrophilic, but can stabilize emulsions by adsorbing to droplets (Pickering emulsion) and via network formation.[20,21] When as little as 0.0011 wt% MC was included, the interfacial tension fell to the value observed for a suspension containing only MC at that concentration (ca 22 mN m^{-1}), and suspensions with higher concentrations of MC had interfacial tensions of ~20 mN m^{-1} regardless of whether CNF was present. Thus unlike CNC,[15] CNF did not compromise the ability of MC to stabilize a water–dodecane interface, even at very low MC concentrations. Moreover, a dilute suspension of CNF had an average ζ-potential of –54 mV, whereas the value for a related suspension that also contained MC (m_{MC}/m_{CNF} = 3.7, the ratio used to produce the wet foams described here) was –26 mV, and clearly showed a bimodal distribution. Thus MC did not shield the surface charge on CNF. TEM images of a sample made from a dilute CNF suspension (Figure 1b) showed networks of entangled fibres as well as some isolated fibres, whereas those of a sample that also contained MC (m_{MC}/m_{CNF} = 3.7, Figure 1c) showed similar networks, as well as large dark regions that we attribute to methylcellulose. Thus we do not see evidence that CNF and MC interact strongly in dilute aqueous suspension.

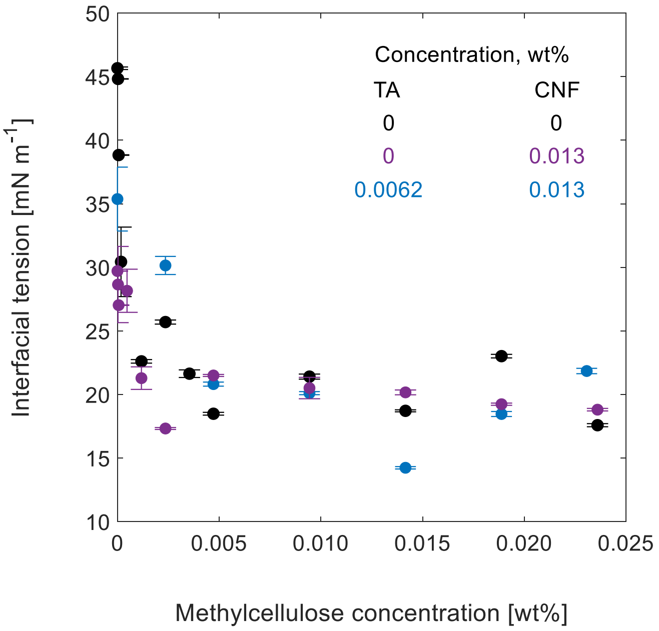

Figure S1. Interfacial tension between an aqueous drop and n-dodecane for suspensions of varying MC concentration. When both CNF and TA are present, they are present in the same ratio used in the foam synthesis.

A dilute suspension containing MC, CNF (m_{MC}/m_{CNF} = 3.7), and a small amount of TA ($m_{TA}/m_{(MC+CNF)}$ = 0.001) contained particles with a more neutral ζ-potential than a suspension containing only MC and CNF, likely due to partial protonation of the carboxylates on CNF by TA. Further, a suspension of CNF and TA with a very small amount of MC had a higher interfacial tension against dodecane than a similar suspension without TA (Figure S1). TA interacts with both cellulose[22] and methylcellulose[23] via hydrophobic interactions (primarily, in the cellulose case), and the very small amount of MC may have interacted preferentially with TA. In the case of our wet foams however, TA was added to a preformed foam supported by MC and CNF;

thus, the MC and CNF were already organized at the air–water interface[24] and in a network in the suspended phase. Such foams are stabilized by the intertwined gel-forming networks of CNF[25] and the surface activity of the MC,[26] and do not expect the tannic acid to cause MC or CNF to desorb from the air–water interface.

S3. Additional data

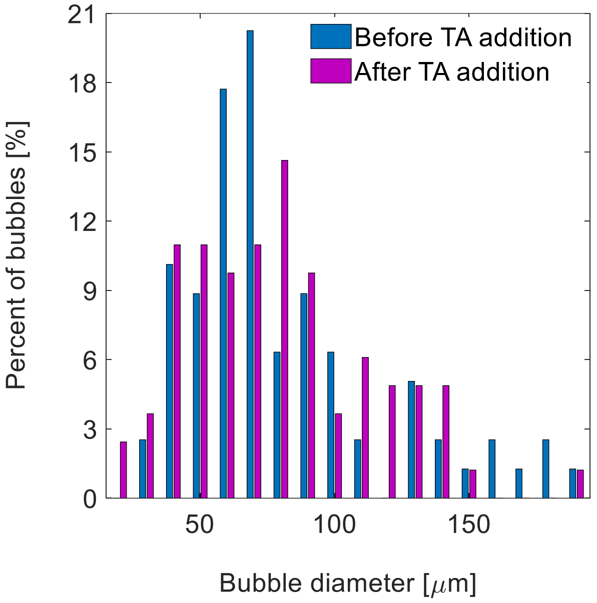

Figure S2. Distribution of bubble sizes in a wet foam (m_{CNF}:m_{MC}:m_{TA} = 21:77:2 and solid content in suspension = 2 wt% when TA addition was complete), before and after TA addition.

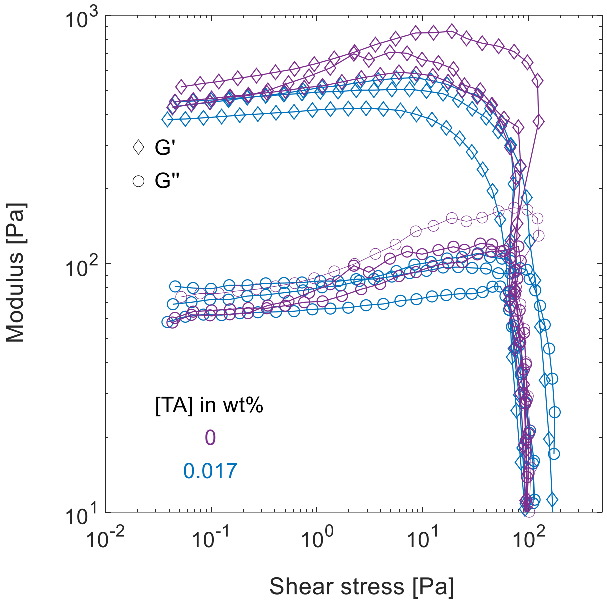

Figure S3. Rheological storage modulus (G ′) and loss modulus (G″) of wet CNF–MC foams, with and without tannic acid. The aqueous phase of both foams contained 0.19 wt% CNF and 0.70 wt% MC.

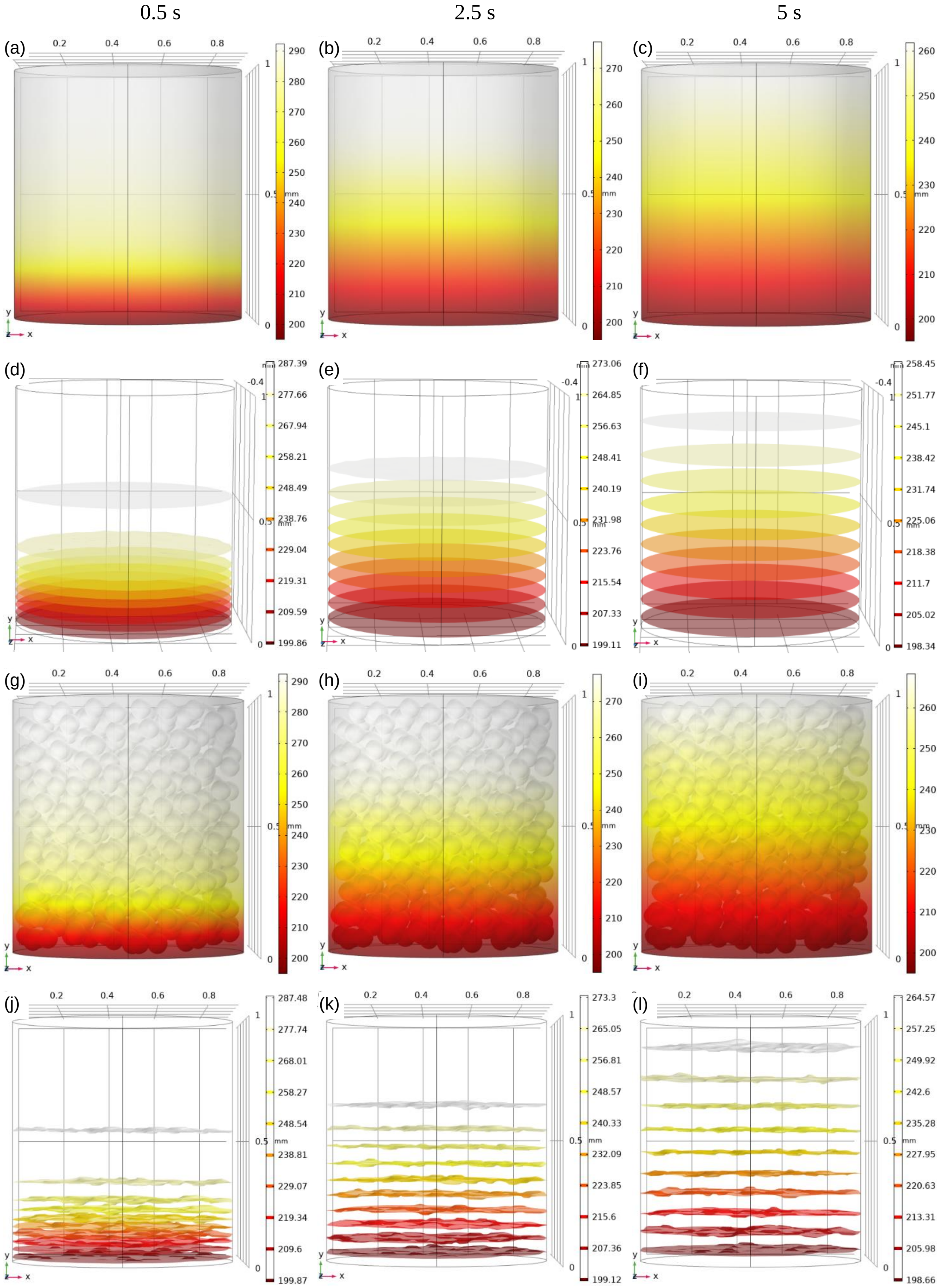

Figure S4. Modelled heat transfer in a cylinder with r = 0.46 mm and h = 1 mm, containing (a–f) water, and (g–l) water and 500 randomly distributed spheres of air with d = 80 µm. Images show temperature distribution (a–c, g–i) and isotherms (d–f, j–l) after the model was run for 0.5 s (a, d, g, j), 2.5 s (b, e, h, k), and 5 s (c, f, i, l).

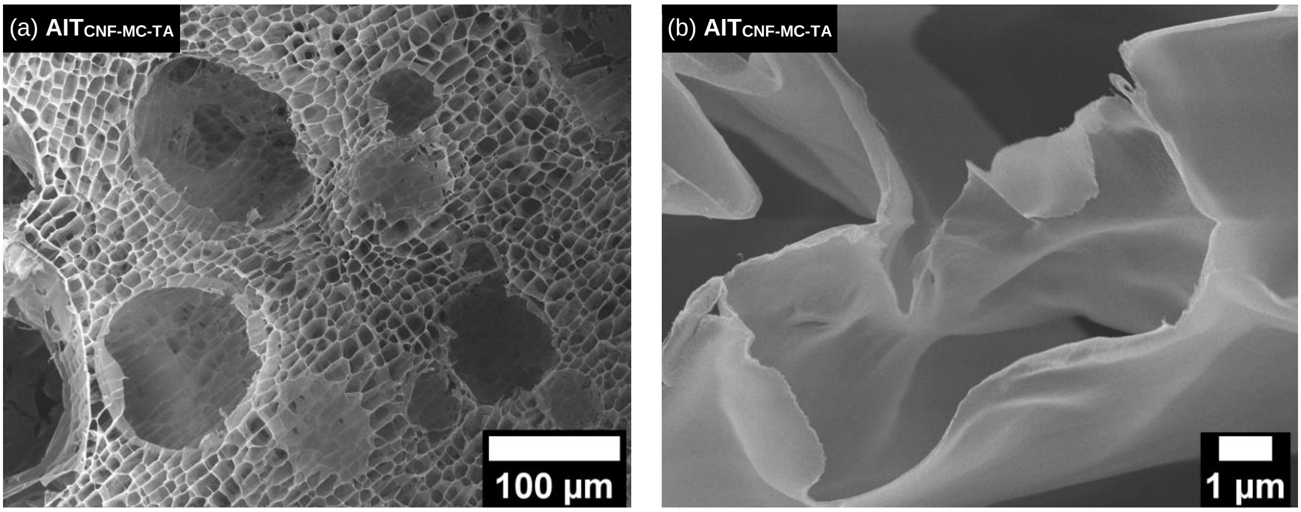

Figure S5. High-magnification scanning electron microscope images of radial cross-sections of the air- and ice-templated **AIT$_{CNF-MC-TA}$** foam. (Figure S5a is also shown as Figure 1d).

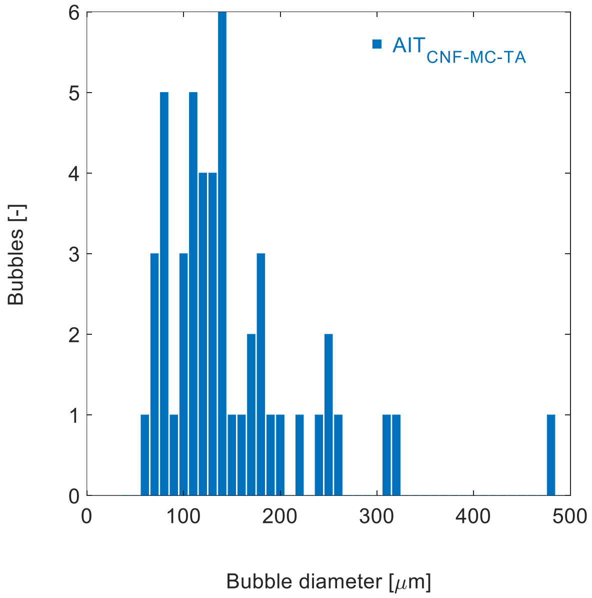

Figure S6. Distribution of spheroid bubble sizes in air-and ice-templated **AIT$_{CNF-MC-TA}$**. Histogram shows the distribution of sizes of 49 bubbles measured on two SEM images.

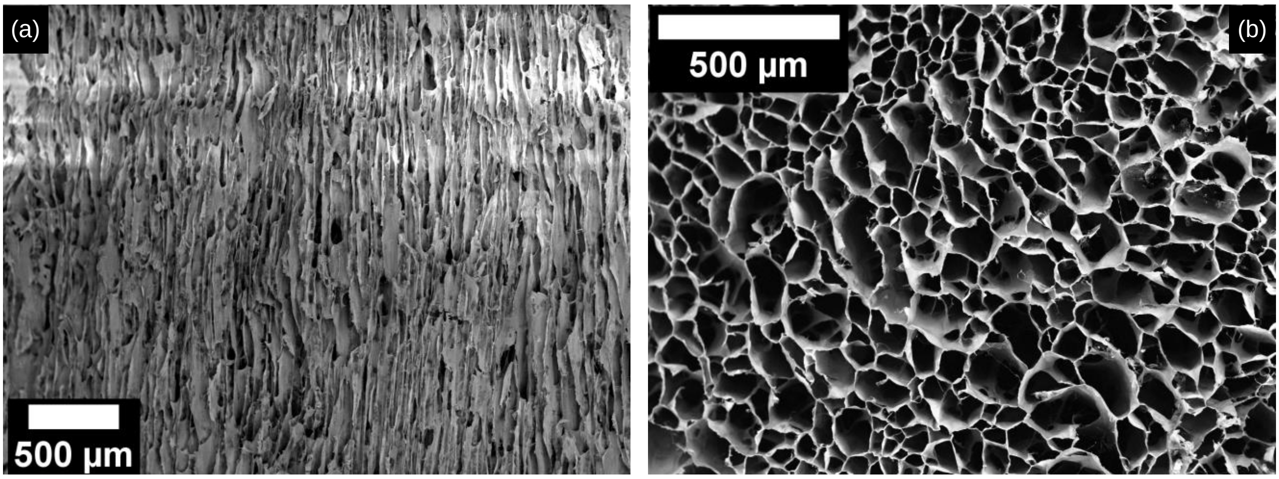

Figure S7. SEM images of **IT$_{CNF}$**, produced by the directional ice-templating of a 0.5 wt% suspension of CNF. (a) Axial cut, and (b) radial cut.

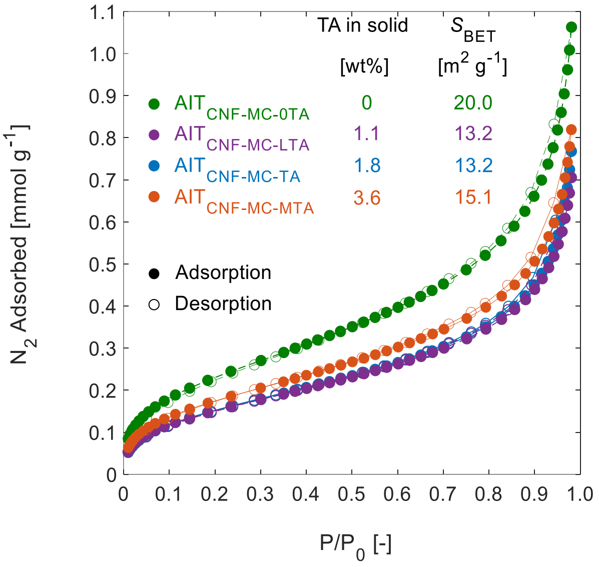

Figure S8. N_2 sorption isotherms of CNF–MC–TA foams with varying TA content, measured at 77 K. m_{MC}/m_{CNF}= 3.7.

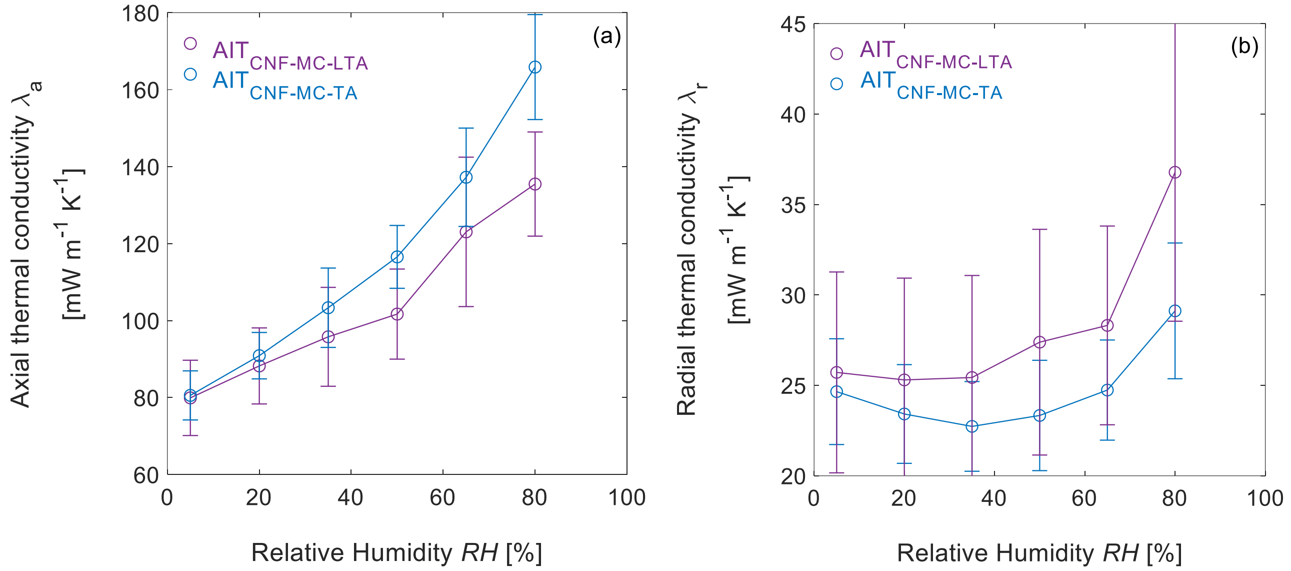

Figure S9. Comparison of the axial (λ_a) and radial (λ_r) thermal conductivities of foams that differ in tannic acid content. In the dry state, **AIT$_{CNF-MC-TA}$** and **AIT$_{CNF-MC-LTA}$** are 1.8 and 1.1 wt% tannic acid. The error bars represent the relative uncertainty of the thermal conductivity from each set of measurements at the relevant relative humidity; see details above.

Table S1. Properties of $HCNF_2$ foams with varying TA contents.[a]

	TA in dry foam [wt%]	ρ[b] [kg m^{-3}]	S_{BET}[c] [m^2 g^{-1}]	Pore wall density[d] [g cm^{-3}]	Porosity[d] [–]	Nanopore[c] Volume [cm^3 g^{-1}]	Compression Properties[e] Young's modulus [kPa]	Compression Properties[e] Energy Absorbed [kJ m^{-3}]
**AIT$_{CNF-MC-0TA}$**	0	16 ± 1	20.0	1.37	0.989	0.033	1170 ± 130	45 ± 7
**AIT$_{CNF-MC-LTA}$**	1.1	11.9 ± 4	13.2	1.38	0.991	0.022	730 ± 200	26 ± 1
**AIT$_{CNF-MC-TA}$**	1.8	18.0 ± 0.5	13.2	1.38	0.987	0.024	1400 ± 300	67 ± 5
**AIT$_{CNF-MC-MTA}$**	3.6	16.8 ± 0.6	15.1	1.39	0.988	0.025	1460 ± 250	57 ± 5

[a] Foams contain TA as per table; the remaining mass is MC and CNF with m_{MC}/m_{CNF} = 3.7. [b] Apparent density, calculated from the dimensions and mass of samples equilibriated at 295 K and 50% relative humidity. [c] Derived from the adsorption branch of the N_2 adsorption isotherm, see Figure S8; S_{BET} = Brunauer–Emmett–Teller surface area, calculated over approx. P/P_0 = 0.05–0.30. [d] See Characterization Methods above for details of calculations. [e] Measured on an average of six or more cylinders (d ~ 1.9 cm; h = 1.5–2.0 cm) at 295 K and 50% RH.

S4. References

(1) Schindelin, J.; Arganda-Carreras, I.; Frise, E.; Kaynig, V.; Longair, M.; Pietzsch, T.; Preibisch, S.; Rueden, C.; Saalfeld, S.; Schmid, B.; Tinevez, J.-Y.; White, D. J.; Hartenstein, V.; Eliceiri, K.; Tomancak, P.; Cardona, A. Fiji: An Open-Source Platform for Biological-Image Analysis. *Nat. Methods* **2012**, *9* (7), 676–682. https://doi.org/10.1038/nmeth.2019.

(2) Schneider, C. A.; Rasband, W. S.; Eliceiri, K. W. NIH Image to ImageJ: 25 Years of Image Analysis. *Nat. Methods* **2012**, *9* (7), 671–675. https://doi.org/10.1038/nmeth.2089.

(3) COMSOL Multiphysics. COMSOL AB: Stockholm, Sweden.

(4) MathWorks. Matlab. 2020.

(5) Kwiatek, G. DXFLib. 2021.

(6) 3D-Convert https://3d-convert.com/en/convert/dxf-to-stl.html.

(7) Püspöki, Z.; Storath, M.; Sage, D.; Unser, M. Transforms and Operators for Directional Bioimage Analysis: A Survey BT - Focus on Bio-Image Informatics; De Vos, W. H., Munck, S., Timmermans, J.-P., Eds.; Springer International Publishing: Cham, 2016; pp 69–93. https://doi.org/10.1007/978-3-319-28549-8_3.

(8) Rezakhaniha, R.; Agianniotis, A.; Schrauwen, J. T. C.; Griffa, A.; Sage, D.; Bouten, C. V. C.; van de Vosse, F. N.; Unser, M.; Stergiopulos, N. Experimental Investigation of Collagen Waviness and Orientation in the Arterial Adventitia Using Confocal Laser Scanning Microscopy. *Biomech. Model. Mechanobiol.* **2012**, *11* (3), 461–473. https://doi.org/10.1007/s10237-011-0325-z.

(9) Brunauer, S.; Emmett, P. H.; Teller, E. Adsorption of Gases in Multimolecular Layers. *J. Am. Chem. Soc.* **1938**, *60* (2), 309–319. https://doi.org/10.1021/ja01269a023.

(10) Barrett, E. P.; Joyner, L. G.; Halenda, P. P. The Determination of Pore Volume and Area Distributions in Porous Substances. I. Computations from Nitrogen Isotherms. *J. Am. Chem. Soc.* **1951**, *73* (1), 373–380. https://doi.org/10.1021/ja01145a126.

(11) Apostolopoulou-Kalkavoura, V.; Hu, S.; Lavoine, N.; Garg, M.; Linares, M.; Munier, P.; Zozoulenko, I.; Shiomi, J.; Bergström, L. Humidity-Dependent Thermal Boundary Conductance Controls Heat Transport of Super-Insulating Nanofibrillar Foams. *Matter* **2021**, *4* (1), 276–289. https://doi.org/10.1016/j.matt.2020.11.007.

(12) Apostolopoulou-Kalkavoura, V.; Munier, P.; Dlugozima, L.; Heuthe, V.-L.; Bergström, L. Effect of Density, Phonon Scattering and Nanoporosity on the Thermal Conductivity of Anisotropic Cellulose Nanocrystal Foams. *Sci. Rep.* **2021**, *11*, 18685. https://doi.org/10.1038/s41598-021-98048-y.

(13) Trofimov, A. A.; Atchley, J.; Shrestha, S. S.; Desjarlais, A. O.; Wang, H. Evaluation of Measuring Thermal Conductivity of Isotropic and Anisotropic Thermally Insulating Materials by Transient Plane Source (Hot Disk) Technique. *J. Porous Mater.* **2020**, *27* (6), 1791–1800. https://doi.org/10.1007/s10934-020-00956-3.

(14) Hynninen, V.; Hietala, S.; McKee, J. R.; Murtomäki, L.; Rojas, O. J.; Ikkala, O.; Nonappa. Inverse Thermoreversible Mechanical Stiffening and Birefringence in a Methylcellulose/Cellulose

Nanocrystal Hydrogel. *Biomacromolecules* **2018**, *19* (7), 2795–2804. https://doi.org/10.1021/acs.biomac.8b00392.

(15) Hu, Z.; Patten, T.; Pelton, R.; Cranston, E. D. Synergistic Stabilization of Emulsions and Emulsion Gels with Water-Soluble Polymers and Cellulose Nanocrystals. *ACS Sustain. Chem. Eng.* **2015**, *3* (5), 1023–1031. https://doi.org/10.1021/acssuschemeng.5b00194.

(16) Kedzior, S. A.; Dube, M. A.; Cranston, E. D. Cellulose Nanocrystals and Methyl Cellulose as Costabilizers for Nanocomposite Latexes with Double Morphology. *ACS Sustain. Chem. Eng.* **2017**, *5* (11), 10509–10517. https://doi.org/10.1021/acssuschemeng.7b02510.

(17) Eronen, P.; Junka, K.; Laine, J.; Österberg, M. Interaction between Water-Soluble Polysaccharides and Native Nanofibrillar Cellulose Thin Films. *BioResources* **2011**, *6* (4), 4200–4217.

(18) Sundman, O. Adsorption of Four Non-Ionic Cellulose Derivatives on Cellulose Model Surfaces. *Cellulose* **2014**, *21* (1), 115–124. https://doi.org/10.1007/s10570-013-0105-2.

(19) Saito, T.; Nishiyama, Y.; Putaux, J.-L.; Vignon, M.; Isogai, A. Homogeneous Suspensions of Individualized Microfibrils from TEMPO-Catalyzed Oxidation of Native Cellulose. *Biomacromolecules* **2006**, *7* (6), 1687–1691. https://doi.org/10.1021/bm060154s.

(20) Goi, Y.; Fujisawa, S.; Saito, T.; Yamane, K.; Kuroda, K.; Isogai, A. Dual Functions of TEMPO-Oxidized Cellulose Nanofibers in Oil-in-Water Emulsions: A Pickering Emulsifier and a Unique Dispersion Stabilizer. *Langmuir* **2019**, *35* (33), 10920–10926. https://doi.org/10.1021/acs.langmuir.9b01977.

(21) Gestranius, M.; Stenius, P.; Kontturi, E.; Sjöblom, J.; Tammelin, T. Phase Behaviour and Droplet Size of Oil-in-Water Pickering Emulsions Stabilised with Plant-Derived Nanocellulosic Materials. *Colloids Surfaces A Physicochem. Eng. Asp.* **2017**, *519*, 60–70. https://doi.org/10.1016/j.colsurfa.2016.04.025.

(22) Tang, H. R.; Covington, A. D.; Hancock, R. A. Structure–Activity Relationships in the Hydrophobic Interactions of Polyphenols with Cellulose and Collagen. *Biopolymers* **2003**, *70* (3), 403–413. https://doi.org/10.1002/bip.10499.

(23) Patel, A. R.; Seijen ten-Hoorn, J.; Hazekamp, J.; Blijdenstein, T. B. J.; Velikov, K. P. Colloidal Complexation of a Macromolecule with a Small Molecular Weight Natural Polyphenol: Implications in Modulating Polymer Functionalities. *Soft Matter* **2013**, *9* (5), 1428–1436. https://doi.org/10.1039/C2SM27200H.

(24) Voisin, H. P.; Gordeyeva, K.; Siqueira, G.; Hausmann, M. K.; Studart, A. R.; Bergström, L. 3D Printing of Strong Lightweight Cellular Structures Using Polysaccharide-Based Composite Foams. *ACS Sustain. Chem. Eng.* **2018**, *6* (12), 17160–17167. https://doi.org/10.1021/acssuschemeng.8b04549.

(25) Cervin, N. T.; Johansson, E.; Benjamins, J.-W.; Wågberg, L. Mechanisms Behind the Stabilizing Action of Cellulose Nanofibrils in Wet-Stable Cellulose Foams. *Biomacromolecules* **2015**, *16* (3), 822–831. https://doi.org/10.1021/bm5017173.

(26) Hu, Z.; Xu, R.; Cranston, E. D.; Pelton, R. H. Stable Aqueous Foams from Cellulose Nanocrystals

and Methyl Cellulose. *Biomacromolecules* **2016**, *17* (12), 4095–4099. https://doi.org/10.1021/acs.biomac.6b01641.
